# Pediatric Nursing and Nasogastric Tube Care: A Bibliometric Analysis of Research Patterns and Themes on a Small Corpus

**DOI:** 10.1155/bmri/5559211

**Published:** 2026-02-09

**Authors:** Shimmaa Mansour Moustafa Mohamed, Gihan Mohamed Mohamed Salem, Raghad Abdulrahman Almushawah, Khalid Mohamed Adam

**Affiliations:** ^1^ Department of Nursing, College of Applied Medical Sciences, University of Bisha, Bisha, Saudi Arabia, ub.edu.sa; ^2^ Florence Nightingale Faculty of Nursing, Midwifery and Palliative Care, King′s College London, London, UK, kcl.ac.uk; ^3^ Department of Medical Laboratory Sciences, College of Applied Medical Sciences, University of Bisha, Bisha, Saudi Arabia, ub.edu.sa

**Keywords:** bibliometric analysis, nasogastric tube, nursing, pediatric, practices

## Abstract

**Background:**

Bibliometrics plays a critical role in supporting decision‐making within the scientific community. It is widely used to evaluate the merit of applications for academic positions and to assess the standing of journals and institutions. This bibliometric analysis is aimed at identifying the growth and trends of nasogastric tube (NGT) research in pediatric settings, evaluating source productivity, and examining the scholarly impact of NGT research.

**Methods:**

We conducted a bibliometric analysis of pediatric NGT nursing literature indexed (search executed 02 March 2024; updated 10 September 2025). Records were retrieved from Scopus and analyzed with Bibliometrix/Biblioshiny (R). To enhance interpretability with a modest corpus, we reported year‐normalized citations, h‐/g‐/m‐indices, and used fractional counting for co‐authorship and country analyses. We mapped co‐citation, bibliographic coupling, co‐word/thematic evolution, and performed robustness checks (parameter thresholds, field restrictions, and time‐window trims).

**Results:**

Seventy‐nine publications were identified. Annual production showed peaks in 2001, 2007, and 2018, with one publication in 2025. The most cited paper was *ASPEN Safe Practices for Enteral Nutrition Therapy* by Boullata et al., which had 335 citations as of 2025. The United States emerged as the leading contributor, followed by the United Kingdom and Canada.

**Conclusion:**

Pediatric NGT research has expanded and diversified over the past 4 decades. The findings highlight the need for sustained investment and stronger international collaboration to improve clinical outcomes and drive innovation in pediatric healthcare. Findings should be interpreted with caution given the niche scope and corpus size, although sensitivity analyses suggested stable high‐level patterns.

**Summary**

*What is Already Known About This Topic?*

• Pediatric nursing requires specialized knowledge and skills, particularly in the care of children requiring nasogastric tube (NGT) placement.
• Existing literature provides insights into NGT care, but comprehensive bibliometric analyses in this area are lacking.
• Evidence regarding the effectiveness of NGT care practices in pediatric settings remains limited.

*What This Paper Adds?*

• This paper presents the first bibliometric analysis of global research on pediatric nursing and NGT care through 2025.
• It identifies the most influential countries, institutions, and authors contributing to the field.
• The findings highlight key thematic areas and gaps, providing a foundation for future studies.

*Implications for Practice and Research*

• Researchers can use these findings to target underexplored areas, strengthening the evidence base for pediatric NGT care.
• Policymakers may apply the results to prioritize funding and support for high‐impact research.
• Educators can incorporate emerging themes and identified knowledge gaps into nursing curricula, preparing a more skilled pediatric nursing workforce.

## 1. Introduction

Nasogastric tube (NGT) feeding is the preferred method of delivering nutrients to children who are unable to swallow, suck, or tolerate oral feeding and medication [1]. Compared with parenteral nutrition, it is safer and more cost‐effective. NGT feeding also improves intestinal membrane immunity by providing the intestine with a favorable environment [2]. It is frequently used in infants with gastrointestinal tract issues, comatose patients, those with increased respiration or in need of mechanical ventilation, premature babies, and patients requiring short‐term feeding [3]. Enteral nutrition (EN) support is widely utilized in both pediatric hospital and community settings. A survey of 63 pediatric facilities revealed that one in four hospitalized children were receiving nutrition through nasogastric, orogastric, or postpyloric feeding tubes [4]. Although the placement of a NGT in pediatric patients is often perceived as a straightforward bedside procedure, each insertion carries a risk of misplacement. Misplaced NGTs can compromise patient safety and may result in severe or even fatal outcomes [5].

Bibliometric analysis is a powerful tool in academic and research environments, enabling the detection and analysis of trends in article and journal performance, collaboration patterns, and the intellectual landscape of specific research fields. This method allows researchers to manage and navigate the large body of literature on particular topics by providing a macroscopic overview of academic work [6–8]. By quantitatively assessing publication histories and the evolution of scientific contributions within a field, bibliometrics offers a comprehensive perspective on the impact and patterns associated with authors, journals, countries, and institutions. It also facilitates the identification of leading contributors and influential works [9, 10].

Furthermore, bibliometric methods reveal the multidisciplinary nature of research themes by examining the range of journals and subject categories publishing on a given topic [11, 12]. In addition to assessing academic contributions, bibliometrics supports decision‐making within the scientific community, being widely used to evaluate applications for academic positions and to determine the standing of journals and institutions. Such information is crucial for policymakers and funding agencies when allocating resources [13]. Overall, bibliometric analysis not only uncovers existing research dynamics but also highlights potential gaps in knowledge, geographically and thematically, making it invaluable for shaping future research directions.

Recent bibliometric reviews in nursing science highlight persistent growth in global nursing research but also fragmentation across specialties [6, 7]. In pediatric nursing, bibliometric work has mapped themes in neonatal care, feeding practices, and technology integration [8]. However, no bibliometric analysis has synthesized research specifically on NGT care in pediatric contexts. This gap reinforces the significance of the current study.

Therefore, the aims of this study are to: (1) identify the growth and trends of NGT research in pediatric settings; (2) evaluate sources′ productivity; and (3) examine scholarly scientific performance impact in NGT research.

## 2. Methodology

### 2.1. Database and Search Strategy

We searched Scopus using: TITLE‐ABS‐KEY((“pediatric∗” OR “child∗” OR “infant∗”) AND (“nurs∗” OR “nursing”) AND (“nasogastric tube∗” OR “NGT” OR “enteral feeding tube∗”)).

Language: English. Document types: articles, reviews, conference papers, editorials, letters, and notes.

The initial search was executed on 02 March 2024 and updated on 10 September 2025. After screening for topic relevance, 79 records were included to explore the breadth and characteristics of the literature on NGT management in pediatric settings, specifically focusing on the role of nurses. To accomplish this, a comprehensive search was performed in Scopus, one of the most inclusive peer‐reviewed literature databases.

The search query was structured as follows:

TITLE‐ABS‐KEY((“pediatric∗” OR “child∗” OR “infant∗”) AND “nurs∗” OR “nursing”) AND (“nasogastric tube∗” OR “NGT” OR “enteral feeding tube∗”)).

**Figure 1:**
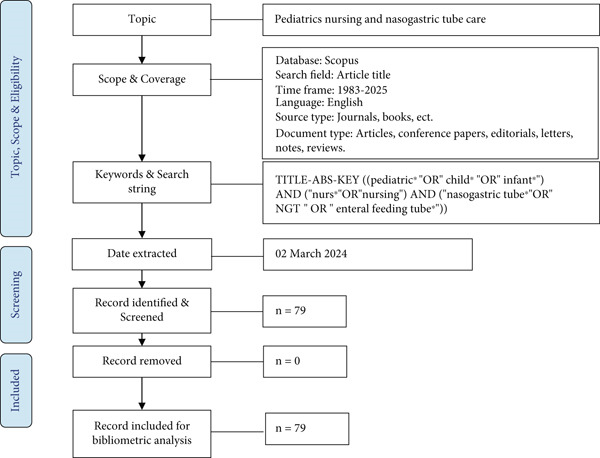
Scopus search flow diagram. Records identified and screened from Scopus; records included in the bibliometric analysis (*n* = 79). Search initially run on 02 March 2024 and updated on 10 September 2025.

This query was designed to retrieve articles that discuss various aspects of NGT usage by nurses in pediatric care environments (Figure 1). The search was first executed on 02 March 2024 and updated on 10 September 2025. After screening for relevance, the updated dataset contained 79 records.

We queried Scopus (Elsevier) using TITLE‐ABS‐KEY to capture pediatric NGT nursing research (initial run 02 March 2024; update 10 September 2025). To mitigate database bias and improve recall in a niche topic, we additionally specify an optional expansion pathway for future updates: Web of Science Core Collection, PubMed/MEDLINE (MeSH), CINAHL, and Embase (Emtree). Synonyms considered include *feeding tube*, *enteral tube*, *nasoenteric*, *orogastric*, and *gavage* combined with pediatric terms and nursing qualifiers. Duplicates would be removed before screening.

Inclusion criteria were English‐language scholarly outputs (articles, reviews, conference papers, editorials, letters, and notes) addressing pediatric NGT care within nursing/clinical practice contexts. Records clearly outside scope (e.g., adult‐only populations; unrelated enteral topics) were excluded.

### 2.2. Indicators and Normalization

Analyses were performed in R (Bibliometrix/Biblioshiny). We report production and impact indicators (total citations (TCs); year‐normalized citations to reduce vintage bias; h‐/g‐/m‐indices). For collaboration and country outputs, we used fractional counting. Networks included co‐authorship, co‐citation, bibliographic coupling, and co‐word mapping with thematic evolution. Layouts used default Bibliometrix algorithms (e.g., association normalization; Fruchterman–Reingold/LinLog layouts with stated thresholds). Parameter choices and thresholds are provided in the supplement.

### 2.3. Sensitivity/Robustness Checks

To assess stability with a modest corpus, we varied: (i) time windows (e.g., 2000–2025 vs. full span), (ii) node thresholds (top‐N vs. frequency cutoffs), and (iii) field restriction (nursing subfield vs. all health sciences). We compared whether top themes, hubs, and country‐level patterns persisted.

### 2.4. Inclusion and Exclusion Criteria

Only articles published in English were included, with no date restrictions applied, to ensure consistency in data interpretation. Eligible document types were research articles, reviews, and conference papers, allowing a comprehensive view of both empirical and theoretical contributions to the field. Editorials, book chapters, and case reports were excluded, as these do not typically provide research‐focused insights central to bibliometric analysis.

### 2.5. Ethical Considerations

This study analyzed bibliographic metadata from published sources only. No human participants, patient‐level data, or identifiable information were involved; therefore, institutional review board/ethics approval was not required under prevailing guidelines. No administrative permissions were needed to access and analyze the aggregated records.

### 2.6. Data Extraction

From the retrieved Scopus dataset, we extracted bibliographic information including publication year, author details, country of origin, keywords, citation counts, and journal names. This dataset formed the basis for the bibliometric assessment, facilitating a multidimensional analysis of publication trends, impacts, and collaborations in the field.

### 2.7. Analytical Techniques

Data were analyzed using R, a statistical programming environment suitable for large datasets and advanced analyses. Within R, the Bibliometrix package was employed, providing a suite of tools for bibliometric analysis such as descriptive statistics, citation analysis, co‐citation analysis, and thematic mapping. Additionally, Biblioshiny, a web‐based interface for Bibliometrix, was used to facilitate visualization and interactive exploration of results. Functions applied included biblioAnalysis(), thematicMap(), co‐word analysis, co‐citation analysis, collaboration mapping, and Lotka′s law modeling. These approaches enabled both descriptive analyses and advanced network/thematic assessments.

## 3. Results

### 3.1. Trends in NGT Research

**Figure 2:**
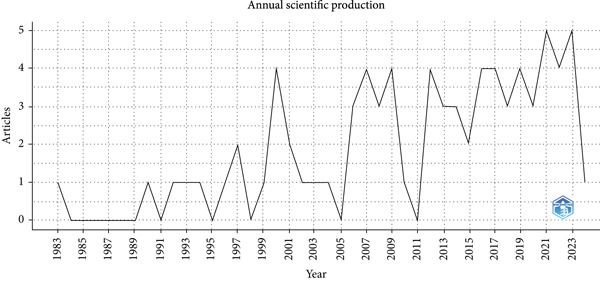
Annual scientific production 1983–2025.

The line graph of annual scientific production (Figure 2) illustrates the fluctuating nature of research output measured by the number of publications (NPs) from 1983 to 2025. A variable pattern is evident, with notable peaks around 2001, 2007, and 2018, followed by periods of decline. One publication was recorded in 2025, extending the timeline of analysis to the most recent year. These fluctuations likely reflect shifts in research priorities, funding availability, and publication practices within the field.

**Figure 3:**
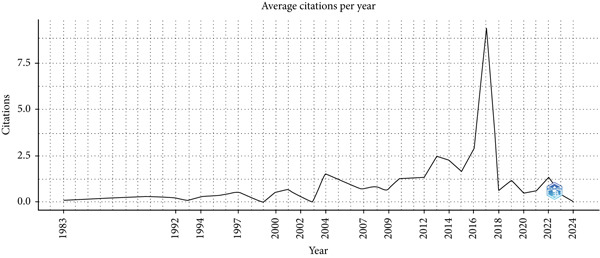
Impact through citations over time.

The citation trend (Figure 3) provides a longitudinal perspective on the impact and recognition of research on pediatric nursing and NGT care between 1983 and 2025. Overall, citations demonstrate an incremental upward trajectory, indicating sustained scholarly engagement. A pronounced spike is observed around 2018, suggesting that articles published during this period attracted exceptional academic attention. The subsequent decline aligns with typical citation life cycles and possible saturation of the topic.

**Figure 4:**
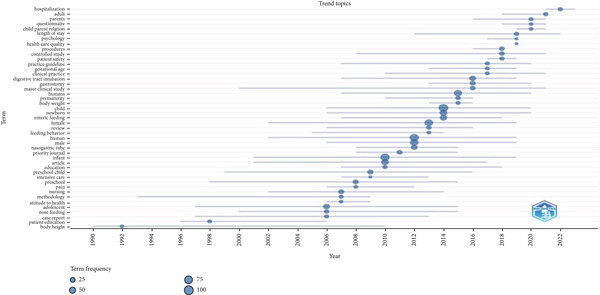
Evolving research themes in pediatric nursing.

The timeline of trending topics (Figure 4) demonstrates the dynamic evolution of research foci over time. Recurring keywords such as “nasogastric tube,” “enteric feeding,” and “newborn” highlight sustained clinical interest, whereas earlier themes such as “body weight” and “patient education” gradually gave way to more specialized clinical terms including “intensive care” and “gestational age.” This temporal distribution underscores the shift from foundational concepts to more advanced and technology‐driven pediatric practices, reflecting evolving clinical challenges and healthcare policies.

### 3.2. Scholarly Scientific Performance

Table 1 categorizes the types of documents analyzed in this bibliometric analysis, totaling 79 publications. Most of these are original research articles, comprising approximately 77.22% of the total. This dominance highlights the central role of primary research in advancing scientific knowledge in this field. Reviews, which account for 16.46%, also make a substantial contribution by synthesizing and consolidating existing evidence. Conference papers, notes, editorials, and letters together represent smaller proportions, yet they provide valuable insights and contribute to specialized discussions within the field.

**Table 1:** Document type.

**Document type**	**Total publications (TP)**	**Percentage (%)**
Article	61	77.22
Review	13	16.46
Conference paper	2	2.53
Note	1	1.27
Editorial	1	1.27
Letter	1	1.27
Total	**79**	**100.00**

**Figure 5:**
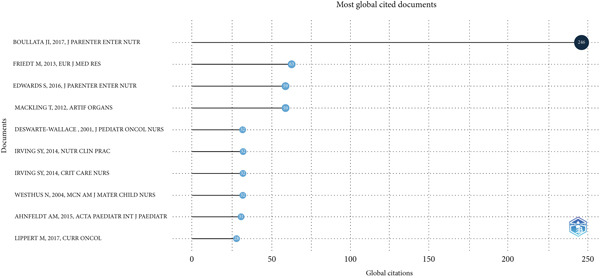
Most influential global publications.

The chart (Figure 5) illustrates the most influential documents in pediatric nursing and related fields, ranked by their global citation counts. At the top of this list, the article by Boullata et al. [14] in the Journal of Parenteral and Enteral Nutrition leads with 335 citations, underscoring its major impact on clinical practice. Other notable contributions include, Edwards et al. [15], with 59 citations, and Friedt and Welsch [16], with 63 citations, both highlighting the importance of nutrition and feeding management in pediatric care. Additional influential works from journals such as Artificial Organs and Critical Care Nurse reflect the diverse scope of research intersecting with pediatric nursing practices. Collectively, these publications map key areas of advancement and sustained research interest shaping pediatric NGT care globally.

Table 2 presents the bibliometric performance of authors in this field, including h‐index, g‐index, m‐index, TCs, NPs, and year of first publication (PY‐start). Lyman B. demonstrates the strongest citation profile with the highest h‐index (5) and g‐index (7) since 2014, supported by 155 citations across seven publications. Guenter P. follows with the largest TC count (270) since 2017, highlighting substantial overall impact despite slightly lower h‐ and g‐index values. Other consistent contributors include Irving S., Kemper C., and Northington L., each active since 2014. In contrast, earlier contributors such as Beckstrand J., Dye J., and Ellett M., despite publishing as early as 1990, display lower citation metrics, indicating more modest long‐term influence.

**Table 2:** Most productive authors in pediatric NGT research.

**Author**	**h-index**	**g-index**	**m-index**	**TC**	**NP**	**PY-start**
Lyman B.	5	7	0.455	155	7	2014
Guenter P.	4	5	0.5	270	5	2017
Irving Sy.	3	3	0.273	78	3	2014
Kemper C.	3	3	0.273	68	3	2014
Northington Ld.	3	3	0.273	50	3	2014
Abdelhadi Ra.	2	2	0.222	18	2	2016
Bartlett Ja.	2	2	0.182	64	2	2014
Beckstrand J.	2	2	0.057	18	2	1990
Dye J.	2	2	0.057	18	2	1990
Ellett M.	2	2	0.057	18	2	1990

*Note:* h‐index: measures both the productivity and citation impact of an author′s publications. g‐index: for quantifying scientific productivity based on publication record. m‐index: is the h‐index divided by the number of years the researcher has been active.

Table 3 provides a detailed overview of the most highly cited documents. The landmark paper “ASPEN Safe Practices for Enteral Nutrition Therapy” by Boullata et al. [14] remains the most impactful, with 335 TCs and an annual citation rate of 30.45. Friedt and Welsch′s update on pediatric endoscopy (2013) has accumulated 63 citations with a steady annual rate of 5.25, whereas Edwards et al. [15] achieved 59 citations with the highest annual rate (6.56) among recent contributions. Mackling et al. [17] on ventricular assist devices demonstrate lasting influence with a normalized TC of 3.42. Earlier studies, including Deswarte‐Wallace et al. [18] on pediatric oncology feeding and Irving et al. [19] on NGT placement, exhibit steady yet modest citation rates, reflecting their specialized focus. Additional works by Ahnfeldt et al. [20] on discharge programs for preterm infants and Lippert et al. [21] on the Hospital at Home Program further highlight the diversification of research, with annual citation rates above 3.0. These patterns illustrate the breadth and evolution of research in pediatric care and EN.

**Table 3:** Highly cited articles.

**Author**	**Title**	**Year**	**Total citations**	**TC per year**	**Normalized TC**
J. Boullata, A. Carrera, L. Harvey, A. Escuro, L. Hudson, A. Mays, C. McGinnis, J. Wessel, S. Bajpai, M. Beebe, T. Kinn, M. Klang, L. Lord, K. Martin, C. Pompeii‐Wolfe, J. Sullivan, A. Wood, A. Malone, P. Guenter	ASPEN Safe Practices for Enteral Nutrition Therapy	2017	335	37.22	1.00
S. Edwards, A. Davis, A. Bruce, H. Mousa, B. Lyman, J. Cocjin, K. Dean, L. Ernst, O. Almadhoun, P. Hyman	Caring for Tube‐Fed Children: A Review of Management, Tube Weaning, and Emotional Considerations	2016	75	7.50	0.22
D.M. Hartman, B. Medoff‐Cooper et al.	Transition to Home after Neonatal Surgery for Congenital Heart Disease	2012	65	4.64	0.19
G. Indramohan, T. Pedigo et al.	Identification of Risk Factors for Poor Feeding Outcomes in Infants with Congenital Heart Disease	2017	57	6.33	0.17
P.A. Kliethermes, M.L. Cross et al.	Transitioning Preterm Infants with Nasogastric Tube Feeding to Oral Feeding: A Randomized Trial	1999	51	1.89	0.15
M.L.C. Cirgin Ellett, M.D. Cohen et al.	Predicting the Insertion Length for Gastric Tubes in Children	2011	43	2.87	0.13
J.C. Evans, D.G. Vogelpohl et al.	Pain Behaviors in LBW Infants Accompany Some “Routine” Procedures	1997	43	1.48	0.13
M. Farrington, S. Lang, et al.	Nasogastric Tube Placement Verification in Pediatric Patients: A Review	2009	41	2.41	0.12
A. Claris‐Appiani, G. Ardissino et al.	Catch‐Up Growth in Children with Chronic Renal Failure	1995	38	1.23	0.11
S.Y. Irving, B. Lyman, L. Northington, J. Bartlett, C. Kemper	Nasogastric Tube Placement and Verification in Children	2014	37	3.08	0.11

**Figure 6:**
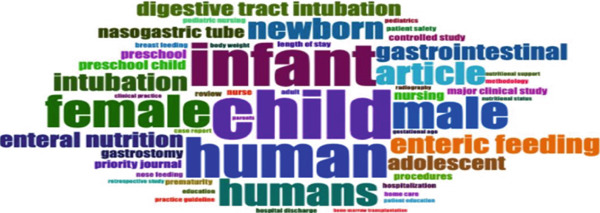
Key research terms in pediatric nasogastric care.

The word cloud (Figure 6) visualizes the most frequent terms in pediatric NGT research. Keywords such as “infant,” “child,” “female,” and “male” emphasize the wide demographic focus of studies. The prominence of “nasogastric tube” alongside “enteric feeding,” “intubation,” and “gastrostomy” reflects sustained attention to procedural and technical aspects of care. Other recurrent terms, including “enteral nutrition,” “nutritional support,” and “hospitalization,” highlight the interdisciplinary dimensions of pediatric NGT research, integrating clinical management with supportive care.

### 3.3. Sources Productivity

**Figure 7:**
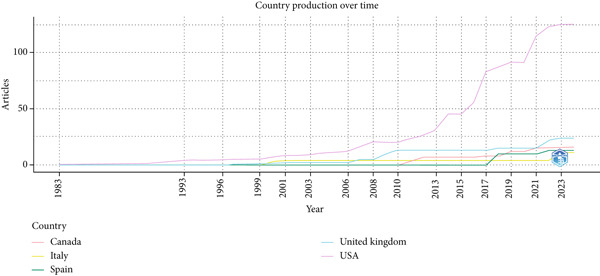
Comparative country output over time.

The temporal analysis of scientific production (Figure 7) reveals a pronounced increase in publications from the United States beginning around 2010, which continued to dominate to 2025. Other countries, including the United Kingdom, Canada, Italy, and Spain, have made steady but comparatively smaller contributions. This trend underscores the central role of the United States while also indicating the gradual expansion of international participation.

**Figure 8:**
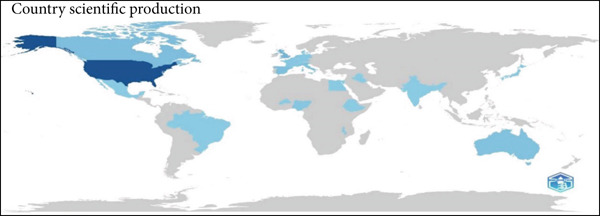
Global distribution of research activity.

The global distribution of research (Figure 8) highlights the geographic breadth of contributions. The United States is the most prolific, followed by the United Kingdom, Germany, and Australia, whereas emerging outputs from Asia and South America demonstrate a widening global footprint. This uneven distribution reflects both the concentration of resources in developed nations and growing attention to NGT research in diverse regions.

**Figure 9:**
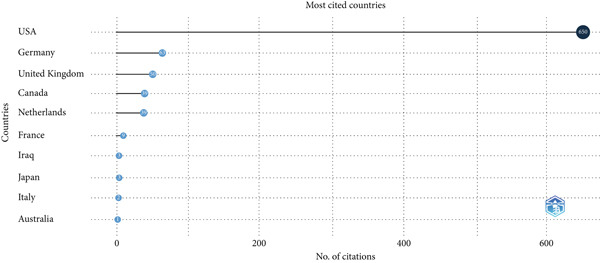
Citation impact by country.

The citation analysis (Figure 9) reinforces the predominance of the United States, which accounts for the majority of citations in this field. Germany, the United Kingdom, and Canada also demonstrate significant citation impact, whereas additional contributions from France, Iraq, Japan, Italy, and Australia indicate growing international recognition, albeit at lower levels.

Table 4 outlines the bibliometric performance of journals contributing to pediatric NGT research. The Journal of Pediatric Nursing and Nutrition in Clinical Practice each hold an h‐index of 4, reflecting their consistent influence. The Journal of Parenteral and Enteral Nutrition, though with a slightly lower h‐index (3), shows the highest overall citation impact with 326 citations since 2016. Specialized outlets such as the Journal of Pediatric Oncology Nursing and Advances in Neonatal Care contribute focused insights, whereas Gastroenterology Nursing and Pediatric Critical Care Medicine offer narrower but valuable perspectives. This distribution highlights both the diversity of research dissemination and the prominence of nutrition‐focused journals in shaping the field.

**Table 4:** Key journals publishing pediatric NGT research.

**Journal**	**h-index**	**g-index**	**m-index**	**TC**	**NP**	**PY-start**
Journal of Parenteral and Enteral Nutrition	**6**	**6**	**0.150**	**490**	**6**	1986
Nutrition in Clinical Practice	**6**	**6**	**0.162**	**91**	**6**	1989
Journal of Pediatric Nursing	**5**	**7**	**0.250**	**119**	**7**	**2006**
JOGNN—Journal of Obstetric, Gynecologic, and Neonatal Nursing	**4**	**4**	**0.138**	**117**	**4**	**1997**
Nursing Times	**3**	**3**	**0.143**	**42**	**3**	**2005**
American Journal of Critical Care	**3**	**3**	**0.188**	**26**	**3**	**2010**
Neonatal Network	**2**	**2**	**0.065**	**74**	**2**	**1995**
Journal for Specialists in Pediatric Nursing	**2**	**3**	**0.118**	**50**	**3**	**2009**
British Journal of Nursing	**2**	**3**	**0.069**	**23**	**3**	**1997**
Journal of Neonatal Nursing	**2**	**2**	**0.133**	**22**	**2**	**2011**

*Note:* h‐index: measures both the productivity and citation impact of the articles published in that journal. g‐index: quantifies a journal′s scientific productivity based on its publication record. m‐index: the h‐index divided by the number of years since the journal started publishing.

**Figure 10:**
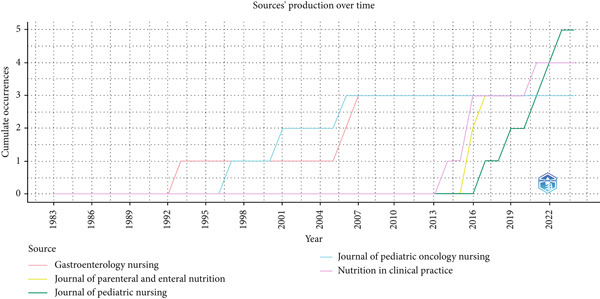
Publication trends in key journals.

The cumulative output of publications by key journals (Figure 10) shows marked increases for Nutrition in Clinical Practice and the Journal of Parenteral and Enteral Nutrition beginning in the early 2010s, reflecting the growing importance of clinical nutrition in pediatric care. The Journal of Pediatric Nursing has also demonstrated steady growth since its inception, whereas Gastroenterology Nursing and the Journal of Pediatric Oncology Nursing show more gradual contributions. Together, these trends underscore the expanding and dynamic nature of journal outlets in disseminating pediatric NGT research.

**Figure 11:**
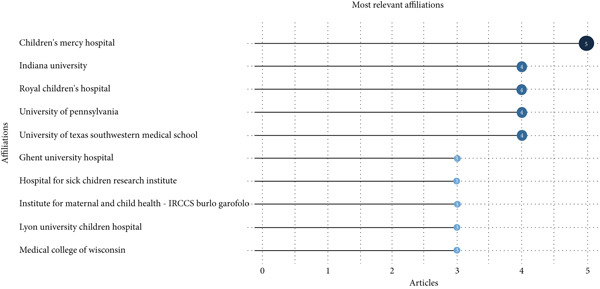
Top institutions in NGT research.

**Figure 12:**
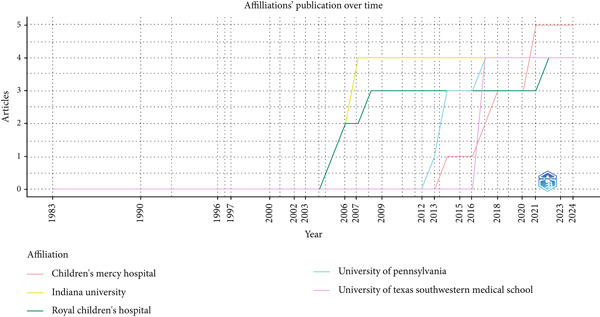
Institutional publication trends.

Institutional productivity analysis (Figure 11) shows that Children′s Mercy Hospital is the most prolific contributor, with five publications. Indiana University, the Royal Children′s Hospital, the University of Pennsylvania, and the University of Texas Southwestern Medical School each contributed four publications, whereas Ghent University Hospital and the Hospital for Sick Children Research Institute produced three each. Temporal patterns indicate phases of growth, with notable surges from 2003 onward, reflecting increased research capacity and sustained institutional engagement in pediatric NGT research (see Figure 12 for the institutional publication trends over time).

### 3.4. Robustness Checks

Key structures (leading sources, recurring themes around verification/safety, nutrition outcomes, and family/caregiver education) were stable across reasonable threshold and window variations. Year‐normalization attenuated age effects while preserving relative ranking of recent influential works. Fractional counting reduced over‐attribution to multicountry consortia without changing the leading contributor set.

### 3.5. Data Availability

The bibliometric dataset was retrieved from Scopus. Processed data underlying the figures and tables (e.g., annual counts, top‐cited documents, and source/journal metrics) are available from the corresponding author upon reasonable request.

### 3.6. Funding Trends

Funding acknowledgments revealed that major sponsors included international agencies such as the National Institutes of Health (United States) and the European Commission, along with national institutions including Saudi research bodies. Countries with higher levels of research investment demonstrated greater productivity, confirming the strong link between funding availability and scientific output. These findings emphasize the critical role of targeted and sustained funding programs in shaping bibliometric patterns and supporting the global advancement of pediatric NGT research.

### 3.7. Discussion

This bibliometric analysis provides an in‐depth overview of pediatric nursing research with a particular emphasis on NGT care, highlighting the dynamic and evolving nature of this field. By integrating bibliometric mapping, bibliometric laws, and thematic synthesis, the study moves beyond descriptive counts to reveal key patterns, contributors, and research gaps.

### 3.8. Research Output and Citations

Analysis of annual scientific production (Figure 2) shows marked fluctuations in research output from 1983 to 2025, with notable peaks in 2001, 2007, and 2018. These peaks correspond with increased citation activity (Figure 3), suggesting that periods of intensive research tend to generate influential work that shapes academic discourse. The observed pattern reflects the typical citation life cycle in which bursts of productivity are followed by heightened recognition. This underlines the importance of sustaining research momentum to ensure continued scholarly and clinical advancements.

### 3.9. Scholarly Scientific Performance and Document Types

The dominance of original research articles (76.92%) (Table 1) emphasizes that primary research is the main driver of scientific advancement in pediatric NGT care. Reviews, which comprise 16.66%, complement this by consolidating evidence and guiding practice. Conference papers, notes, editorials, and letters, though fewer in number, provide focused insights and extend dialog on specialized topics. Bibliometric laws further explain these distributions: Lotka′s law shows that a small group of prolific authors contributes disproportionately to the literature, whereas Bradford′s law confirms concentration of publications in a few core journals such as the *Journal of Pediatric Nursing* and the *Journal of Parenteral and Enteral Nutrition (JPEN)*. Zipf′s law highlights recurring keywords (“nasogastric tube,” “infant,” and “enteral feeding”), and Price′s law suggests exponential growth between 2000 and 2020, followed by a relative plateau.

### 3.10. Most Influential Global Publications

The most highly cited paper is *ASPEN Safe Practices for Enteral Nutrition Therapy* by Boullata et al. [14], which has accrued 335 citations, reflecting its global significance as a reference for best practices in EN (Figure 5, Table 3). This paper set benchmarks for clinical safety and quality in NGT care. Other influential works include Friedt and Welsch [16], with 63 citations, and Edwards et al. [15], with 59 citations, both maintaining high annual citation rates that signal their continued relevance. These findings confirm that highly cited publications shape standards of care, guide clinical protocols, and direct future research agendas.

### 3.11. Author Contributions and Impact

The bibliometric analysis of authors (Table 2) highlights variation in productivity and impact. Lyman B., with the highest h‐index (5) and g‐index (7) since 2014, demonstrates strong and consistent scholarly influence. Guenter P., despite a slightly lower h‐index, has achieved the highest TCs (270) since 2017, underscoring substantial impact. Other contributors such as Irving S., Kemper C., and Northington L. show steady productivity, whereas earlier authors including Beckstrand J., Dye J., and Ellett M. exhibit a modest long‐term impact despite starting their contributions as early as 1990. These results illustrate how both recent and earlier contributions continue to shape the knowledge base.

### 3.12. Highly Cited Articles

Table 3 provides further evidence of the most impactful publications. Boullata et al. [14] stands out not only in TCs but also in annual citation rate (30.45), reinforcing its sustained influence. Edwards et al. [15], with an annual citation rate of 6.56, has served as a practical guide in clinical care of tube‐fed children. Mackling et al. [17], despite earlier publication, maintains a high‐normalized TC (3.42), demonstrating enduring relevance. Other specialized contributions, including Deswarte‐Wallace et al. [18] on oncology patients and Irving et al. [19] on verification practices, highlight the diversity and specialized niches within pediatric NGT research. Additional contributions such as Ahnfeldt et al. [20] on discharge programs for preterm infants and Lippert et al. [21] on the Hospital at Home program demonstrate meaningful impact in clinical care.

### 3.13. Key Research Terms in Pediatric Nasogastric Care

The word cloud (Figure 6) confirms recurring research priorities. Demographic descriptors such as “infant” and “child” reflect the breadth of populations studied. Core terms including “nasogastric tube,” “enteric feeding,” “intubation,” and “gastrostomy” reveal the technical focus of this body of work. Additional terms like “enteral nutrition,” “nutritional support,” and “hospitalization” underscore the importance of interdisciplinary care and supportive frameworks in pediatric NGT management.

### 3.14. Geographic Disparities and Contributions

The geographical distribution (Figures 7 and 8) underscores the prominent role of the United States in both research production and citations. This reflects a strong research infrastructure and sustained funding that drive innovations in pediatric NGT practices. Other countries, notably the United Kingdom, Canada, and Germany, have made meaningful contributions, though at comparatively lower levels. Emerging outputs from Asia and South America highlight the global relevance of this field and suggest potential growth in underrepresented regions.

### 3.15. Citation Impact by Country

Citation analysis (Figure 9) reinforces the global impact of research emanating from the United States, which leads in both volume and citations. Germany and the United Kingdom also show substantial citation influence, whereas countries such as France, Iraq, Japan, Italy, and Australia provide smaller yet significant contributions. These findings reveal that while productivity is concentrated, the influence of pediatric NGT research is increasingly international, shaping practice standards and informing care worldwide.

### 3.16. Influential Journals and Institutional Contributions

Analysis of journals (Table 4 and Figures 10 and 11) confirms that the *Journal of Pediatric Nursing* and *Nutrition in Clinical Practice* are key outlets for dissemination, both showing growth in output and influence. The *Journal of Parenteral and Enteral Nutrition*, with 326 citations since 2016, demonstrates its pivotal role in nutrition‐focused research. Institutionally, Children′s Mercy Hospital emerges as the most prolific contributor, followed by Indiana University, the Royal Children′s Hospital, the University of Pennsylvania, and the University of Texas Southwestern Medical School. These institutions show phases of accelerated growth, particularly post‐2003, reflecting increased capacity and targeted investment.

### 3.17. Institutional Publication Trends

Institutional analysis further illustrates shifts in productivity over time (Figure 12). Children′s Mercy Hospital and Indiana University maintained steady outputs until the early 2000s, after which production rose markedly. The Royal Children′s Hospital exhibited significant surges in 2003 and 2013, suggesting phases of strategic expansion. The University of Pennsylvania displayed consistent growth until plateauing around 2016, whereas the University of Texas Southwestern Medical School demonstrated later but sustained increases beginning in 2007. These trajectories reflect both institutional priorities and the evolving emphasis on pediatric NGT care.

### 3.18. Evolution of Research Themes

Thematic evolution (Figure 4) highlights shifts from foundational terms such as “body weight” and “patient education” to more specialized areas including “gestational age” and “intensive care.” This reflects changing clinical priorities, the adoption of new medical technologies, and responses to emerging healthcare challenges. Thematic clustering revealed four major knowledge domains: (1) safety and verification methods for tube placement, (2) nutritional outcomes and complications, (3) caregiver and parent education, and (4) innovations in pediatric enteral care. These themes not only highlight current priorities but also identify gaps, particularly the need for standardized verification protocols, multicenter collaborations, and family‐centered interventions.

Although the corpus (*n* = 79) is modest, this reflects the narrow clinical focus of pediatric NGT nursing. We therefore emphasized conservative, normalization‐aware indicators and sensitivity analyses. In niche bibliometrics, a smaller but thematically coherent set can still yield reliable structural signals (e.g., stable thematic clusters and consistent core sources/authors) provided methods are transparent and robustness is demonstrated as done here. We nonetheless recommend continued updates and cross‐database triangulation as the field grows.

### 3.19. Overall

This study mapped pediatric NGT bibliometric trends and revealed persistent gaps in caregiver education, outcome monitoring, and multicenter collaboration. The findings underscore the need for targeted investment in pediatric enteral research, stronger global collaborations, and integration of evidence into nursing curricula and health policy to ensure a safer and more effective NGT care for children.

## 4. Conclusion

This bibliometric analysis enhances understanding of pediatric NGT care research by systematically mapping historical and contemporary research dynamics. It identified the evolution of research themes, influential contributors, and institutional productivity, thereby providing a comprehensive overview of the field. By highlighting both strengths and gaps, this study offers a valuable resource for researchers, clinicians, and policymakers, supporting the development of future clinical guidelines, educational curricula, and research agendas in pediatric nursing.

## 5. Limitations

First, the corpus size is limited, consistent with the specificity of pediatric NGT nursing, and results should be interpreted cautiously. Second, although bibliometrics maps structures and trends, it does not evaluate the methodological quality or clinical effectiveness of individual studies. Third, our primary dataset derives from Scopus; while widely used, single‐index reliance may omit items from other platforms. We mitigated these issues by employing year‐normalized metrics, fractional counting, and robustness checks; future updates can expand to multiple databases and non‐English records.

## 6. Future Research

Future investigations should complement bibliometric mapping with qualitative evaluations to assess the clinical applicability and impact of research findings. Expanding analyses to include multiple databases and non‐English literature would also provide a more comprehensive global perspective. Moreover, there is a need for research that strengthens interdisciplinary collaborations, evaluates long‐term patient outcomes, and explores caregiver education and family‐centered interventions in pediatric NGT care.

## Disclosure

All authors have read and approved the final manuscript and agreed to be accountable for its contents.

## Conflicts of Interest

The authors declare no conflicts of interest.

## Author Contributions

**Shimmaa Mansour Moustafa Mohamed:** conceptualization, methodology, formal analysis, supervision, writing – review & editing. **Gihan Mohamed Mohamed Salem:** methodology, writing – original draft, writing – review & editing. **Raghad Abdulrahman Almushawah:** data curation, visualization, writing – original draft, writing – review & editing. **Khalid Mohamed Adam:** data curation, formal analysis, visualization, writing – review & editing.

## Funding

No funding was received for this manuscript.

## Data Availability

Aggregated bibliometric data and analysis scripts are available from the corresponding author upon reasonable request.
